# Elevated IgA antiphospholipid antibodies in healthy pregnant women in Sudan but not Sweden, without corresponding increase in IgA anti-β_2_ glycoprotein I domain 1 antibodies

**DOI:** 10.1177/0961203320908949

**Published:** 2020-02-27

**Authors:** S Elbagir, N A Mohammed, H Kaihola, E Svenungsson, I Gunnarsson, V A Manivel, E Pertsinidou, EM Elagib, M A M Nur, E A Elussein, A Elshafie, H Åkerud, J Rönnelid

**Affiliations:** 1 Department of Immunology, Genetics and Pathology, Uppsala University, Uppsala, Sweden; 2 Faculty of Medical Laboratory Sciences, Al Neelain University, Khartoum, Sudan; 3 Division of Rheumatology, Department of Medicine, Solna, Karolinska Institutet, Karolinska University Hospital, Stockholm, Sweden; 4 Rheumatology Unit, Military Hospital, Omdurman, Sudan; 5 Rheumatology Unit, Alribat University Hospital, Khartoum, Sudan; 6 Khartoum Fertility Center, Academy of Medical Sciences and Technology, Khartoum, Sudan

**Keywords:** Antiphospholipid antibodies, IgA, pregnancy, healthy, Sudan, Africa

## Abstract

**Objective:**

The role of antiphospholipid antibodies (aPL) during apparently normal pregnancy is still unclear. IgA aPL are prevalent in populations of African origin. Our aim was to measure all isotypes of anticardiolipin (anti-CL) and anti–β_2_ glycoprotein I (anti-β_2_GPI) in healthy pregnant and non-pregnant women of different ethnicities.

**Methods:**

Healthy Sudanese pregnant women (*n* = 165; 53 sampled shortly after delivery), 96 age-matched Sudanese female controls and 42 healthy pregnant and 249 non-pregnant Swedish women were included. IgA/G/M anti-CL and anti-β_2_GPI were tested at one time point only with two independent assays in Sudanese and serially in pregnant Swedes. IgA anti-β_2_GPI domain 1 and as controls IgA/G/M rheumatoid factor (RF), IgG anti–cyclic citrullinated peptide 2 (anti-CCP2) and anti–thyroid peroxidase (anti-TPO) were investigated in Sudanese females.

**Results:**

Pregnant Sudanese women had significantly higher median levels of IgA anti-CL, IgA anti-β_2_GPI (*p* < 0.0001 for both antibodies using two assays) and IgM anti-β_2_GPI (both assays; *p* < 0.0001 and 0.008) compared with non-pregnant Sudanese. IgA anti-CL and anti-β_2_GPI occurrence was increased among Sudanese pregnant women compared with national controls. No corresponding increase during pregnancy was found for IgA anti-β_2_GPI domain 1 antibodies. Both IgG anti-CL and IgG control autoantibodies decreased during and directly after pregnancy among Sudanese. Serially followed Swedish women showed no changes in IgA aPL, whereas IgG/M anti-CL decreased.

**Conclusions:**

IgA aPL are increased in Sudanese but not in Swedish women, without corresponding increase in IgA domain 1. Whether due to ethnicity and/or environmental influences the occurrence of IgA aPL during Sudanese pregnancies, and its clinical significance, is yet to be determined.

## Introduction

Antiphospholipid antibodies (aPL) are well-recognized as the most common acquired and treatable risk factor for recurrent fetal loss.^1^ β_2_ glycoprotein I (β_2_GPI) is frequently described as the main culprit antigen in antiphospholipid syndrome (APS) including aPL-induced pregnancy morbidity.^2,3^ As suggested by previous in vivo and in vitro studies, different aPL-mediated mechanisms are involved in the pathogenesis of pregnancy-related complications and fetal loss. These mechanisms include placental tissue thrombosis and inflammation, distorted trophoblastic function and growth and complement activation.^4–6^ Autoantibodies against domain 1 of β_2_GPI (β_2_GPI-D1) in particular have been shown to be associated with thrombotic events as compared with other epitopes.^7,8^

According to the current Sapporo classification criteria APS includes only IgG and IgM but not IgA as isotypes for anticardiolipin (anti-CL) and anti-β_2_GPI testing.^1^ The latest international task force in 2014 found low degree of evidence for including IgA aPL in the classification criteria.^9^ Nevertheless, previous reports have indicated the considerable prevalence and clinical significance of IgA aPL in relation to obstetric adverse events, fetal demise and thrombosis in systemic lupus erythematosus (SLE) and/or APS.^10–15^ Among Caucasian SLE patients there is a clear preponderance for IgG aPL, whereas a parallel IgA aPL preponderance was observed among African American SLE^16,17^ and APS patients.^18^ Importantly, a previous paper from South Africa has reported that SLE patients with secondary APS showed the strongest association with IgA aPL,^19^ and in another study including 73 Kuwaiti women of Arabic ancestry both IgA and IgM but not IgG aPL associated with recurrent spontaneous abortions.^10^ It is noteworthy that IgA aPL was added to the 2012 Systemic Lupus International Collaborating Clinics/American College of Rheumatology criteria for SLE classification.^20^

Pregnancy is a unique physiological and immunological state that is characterized by different biological changes to favor the environment and reception of the fetus.^21^ Changes of immunoglobulin isotype levels during normal pregnancy have been shown in early studies consistently reporting a drop in IgG and fluctuations in IgA and IgM levels as compared with pre-pregnancy state.^22–25^ Previous papers documented decrease of aPL towards the end of pregnancy, with levels mostly within the pre-pregnancy reference range.^26,27^ However, Lynch et al. have demonstrated that high levels of IgG anti-CL at the first antenatal visit before 25 weeks gestation correlated to fetal loss, and in a later longitudinal study they reported significant variations in IgG anti-CL levels during normal pregnancy, but repeated measurements of aPL did not add to risk stratification of these pregnancies.^28,29^

Many reports addressing the role and clinical impact of IgG and IgM aPL in normal pregnancies and in obstetric APS have been published previously.^2,9,28,30^ Data on the prevalence and significance of IgA aPL are, however, limited and almost completely lacking in populations of African origin. Given the special interest of IgA aPL in these populations, our goal was to investigate the occurrence of aPL isotypes during apparently healthy pregnancies in Sudan. As comparators, we also investigated healthy non-pregnant women from Sudan and healthy pregnant and non-pregnant women from Sweden. In Sudanese women we also investigated other autoantibodies: rheumatoid factor (RF) of all three isotypes, IgG anti–cyclic citrullinated peptide 2 (anti-CCP2) and IgG anti–thyroid peroxidase (anti-TPO).

## Patients and methods

### Participants

This study included 165 healthy pregnant and 96 healthy non-pregnant Sudanese women. Fifty-three of the pregnant women were sampled shortly (up to 6 h) after delivery. They were compared with 42 healthy pregnant Swedish women followed serially, and 249 non-pregnant population controls from the Karolinska case-control SLE cohort. All participants gave oral and/or written informed consent to participate in the study that was performed in agreement with the Declaration of Helsinki. Approval was obtained from the Ethical Committees of Alribat University Hospital (11 April 2011) and Omdurman Military Hospital (25 May 2011) for the Sudanese subjects and from the Uppsala and Karolinska University Hospital Ethics Committees for the Swedish subjects.

All Sudanese females were included during 2013–2014. Pregnant women were recruited at Alribat University Hospital and Omdurman Military Hospital in Khartoum. Information about age, parity and gestational age was provided from medical records. Non-pregnant females were university employees and students at Al Neelain University in Khartoum. Median age in years (range) of the pregnant, non-pregnant and delivered Sudanese women were 31 (19–43), 29 (22–43) and 29 (19–39) years, respectively. Among the pregnant females, 16.5%, 21.1% and 62.4% were in the first, second and third trimesters, respectively. Median number of previous pregnancies of the pregnant women including the recently delivered was three. Venous blood was collected from all subjects, immediately centrifuged, then separated, and the serum was stored at −70℃. Serum samples were available from 107 pregnant, 95 non-pregnant and 53 delivered females.

Healthy Swedish pregnant women were enrolled during 2004–2007 in Karlstad, Sweden as previously described.^31^ Median age (range) for the pregnant women was 28 (21–37) years, and 26, 13 and 1 females were in the first, second and third pregnancy, respectively. Plasma samples were available from 38 women and were serially collected at four time points corresponding to each trimester with the last two being at early and late third trimester. As non-pregnant cross-sectional controls for Swedes, we have included 249 population-based females from the Karolinska case-control SLE cohort who have been recruited during 2004–2011.^32,33^

This study was planned as a cross-sectional study; as a consequence of the unexpected immunological findings in the Sudanese cohort, inclusion and further analysis of the Swedish cohort was performed as a comparator.

### Immunological testing

Quantification of autoantibody isotypes IgA, IgG and IgM anti-CL and anti-β_2_GPI was performed using the EliA system based on fluorescence enzyme immunoassay (FEIA) on the Phadia 2500 instrument (Thermo Fisher Scientific, Uppsala, Sweden) according to the manufacturer’s instructions, and the same analyses were repeated for Sudanese females using the Aptiva system based on a particle-based multi-analyte technology (PMAT) (Inova Diagnostics, San Diego, CA, USA, research use only). Among the Sudanese subjects, IgA against β_2_GPI-D1 were analyzed using modified QUANTA Flash β_2_GPI-D1 chemiluminescence assay (BIO-FLASH, Inova Diagnostics, research use only^34^); control autoantibodies IgA, IgG and IgM RF, IgG anti-CCP2 and IgG anti-TPO were determined with FEIA (Thermo Fisher Scientific). Analysis of autoantibodies in plasma and serum specimens yielded similar results using the Thermo Fisher system (internal study from Thermo Fisher Scientific, Phadia GmbH). Lupus anticoagulant test was not performed in the Sudanese cohort due to unfeasibility to conform to sample collection and processing guidelines at the time of recruitment of subjects.

Three cut-offs were evaluated for each aPL isotype: the manufacturers’ suggested values, and the 95th and the 99th percentiles of the respective national non-pregnant women.

### Statistical analysis

For comparisons between quantitative variables between several groups, Kruskal–Wallis test was performed, and whenever significant it was followed by Mann–Whitney *U* test for each pair. Chi-squared test was conducted to test for differences between categorical variables with Fisher’s exact test applied when appropriate. For paired quantitative variables Wilcoxon signed rank test was used. All statistical analyses including national cut-off calculations were conducted using JMP statistical software (SAS Institute, Cary, NC, USA). All *p*-values <0.05 were considered significant.

## Results

**Figure 1:**
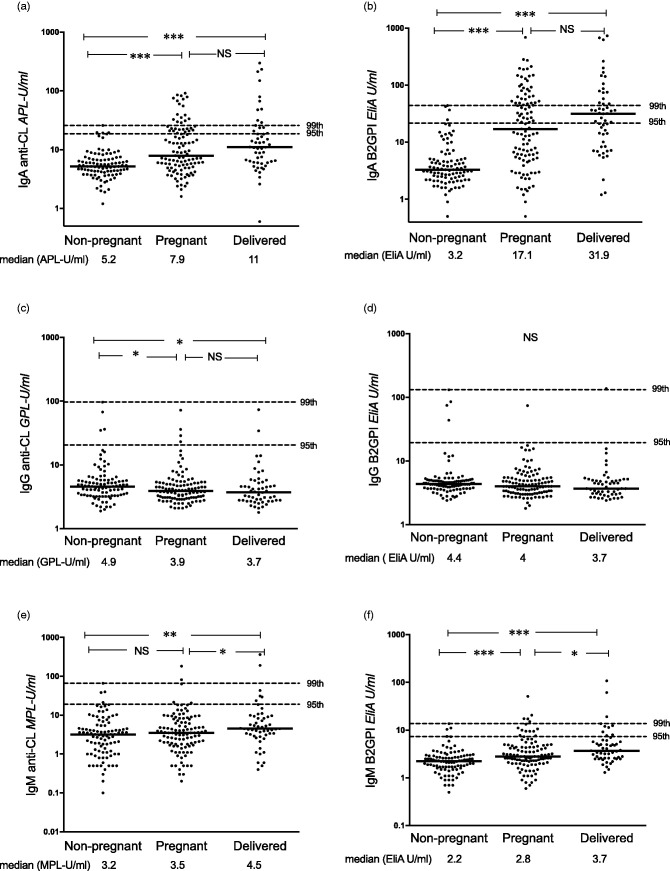
Levels of IgA/G/M anticardiolipin (anti-CL) and anti–β_2_ glycoprotein I (anti-β_2_GPI) antibodies tested with fluorescence enzyme immunoassay among Sudanese women. (a), (c) and (e) IgA/G/M anti-CL and (b), (d) and (f) IgA/G/M anti-β_2_GPI levels among healthy non-pregnant, pregnant and recently delivered females. Horizontal dotted lines represent the 95th and 99th percentiles among the healthy non-pregnant women. Comparisons between individual groups were made for those antibodies showing an overall significant difference between the three groups. *p < 0.05, **p < 0.01, ***p < 0.001, NS: not significant.

**Figure 2:**
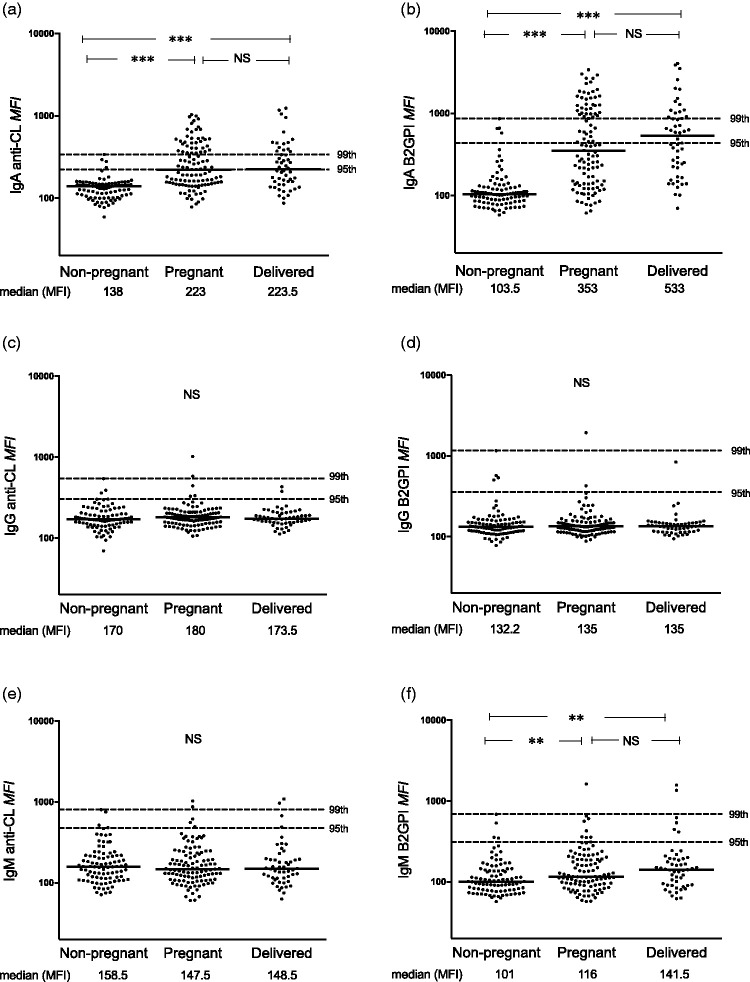
Levels of IgA/G/M anticardiolipin (anti-CL) and anti–β_2_ glycoprotein I (anti-β_2_GPI) antibodies tested with particle-based multi-analyte technology among Sudanese women. (a), (c) and (e) IgA/G/M anti-CL and (b), (d) and (f) IgA/G/M anti-β_2_GPI levels among healthy non-pregnant, pregnant and recently delivered females. Horizontal dotted lines represent the 95th and 99th percentiles among the healthy non-pregnant women. Median fluorescence intensity (MFI) is a raw measurement unit and not a final calibrated unit. Comparisons between individual groups were made for those antibodies showing an overall significant difference between the three groups. *p < 0.05, **p < 0.01, ***p < 0.001, NS: not significant.

Median levels of IgA anti-CL and IgA anti-β_2_GPI (*p* < 0.0001 for both antibodies using two assays) and IgM anti-β_2_GPI (both assays; *p* < 0.0001 and 0.008) were higher among pregnant than among non-pregnant Sudanese women. These differences were evident also when comparing the recently delivered with non-pregnant women (Figures 1 and 2). Median IgA aPL levels in post-partum females were higher compared with pregnant women, although not statistically significant. IgG anti-CL levels on the other hand were lower during pregnancy and shortly after childbirth as compared with non-pregnant women (*p* = 0.02 and *p* = 0.01; Figure 1). Median levels did not differ for any aPL between the 42 pregnant women in the last gestation month and post-partum women (data not shown). Very similar results were obtained using FEIA (Figure 1) and PMAT (Figure 2). Using all three cut-offs, the occurrence of IgA anti-CL and anti-β_2_GPI tested by both assays was significantly higher in pregnant compared with non-pregnant Sudanese women (Tables 1 and 2), whereas IgM anti-β_2_GPI showed higher prevalence among pregnant females when using the 95th Sudanese national cut-off in FEIA (Table 1), but not using PMAT.

**Table 1:** Prevalence of anticardiolipin and anti–β_2_ glycoprotein I isotypes tested with fluorescence enzyme immunoassay among healthy NP, pregnant and RD Sudanese females.

	NP *n* = 95 *n* (%)	Pregnant *n* = 107 *n* (%)	RD *n* = 53 *n* (%)	*p*-value (overall)	*p*-value (NP-pregnant)	*p*-value (NP-RD)	*p*-value (pregnant-RD)
IgA							
CL 95th	4 (4.2)	31 (28.9)	15 (28.3)	**<0.0001**	**<0.0001**	**<0.0001**	0.9
CL 99th	0 (0)	17 (15.9)	11 (20.7)	**<0.0001**	**<0.0001**	**<0.0001**	0.4
CL Phadia	1 (1)	30 (28)	14 (26.4)	**<0.0001**	**<0.0001**	**<0.0001**	0.8
β_2_GPI 95th	4 (4.2)	51 (47.7)	31 (58.5)	**<0.0001**	**<0.0001**	**<0.0001**	0.2
β_2_GPI 99th	0 (0)	35 (32.7)	17 (32.1)	**<0.0001**	**<0.0001**	**<0.0001**	0.9
β_2_GPI Phadia	15 (15.8)	64 (59.8)	40 (75.5)	**<0.0001**	**<0.0001**	**<0.0001**	0.05
IgG							
CL 95th	4 (4.2)	4 (3.7)	2 (3.8)	0.9			
CL 99th	0 (0)	0 (0)	0 (0)	NA			
CL Phadia	2 (2.1)	1 (0.9)	1 (1.9)	0.9			
β_2_GPI 95th	4 (4.2)	1 (0.9)	1 (1.9)	0.3			
β_2_GPI 99th	0 (0)	0 (0)	1 (1.9)	0.1			
β_2_GPI Phadia	8 (8.4)	9 (8.4)	4 (7.6)	0.9			
IgM							
CL 95th	4 (4.2)	6 (5.6)	6 (11.3)	0.2			
CL 99th	0 (0)	2 (1.9)	2 (3.8)	0.2			
CL Phadia	1 (1)	3 (2.9)	3 (5.7)	0.3			
β_2_GPI 95th	4 (4.2)	15 (14.0)	10 (18.9)	**0.01**	**0.02**	**0.003**	0.4
β_2_GPI 99th	0 (0)	5 (4.7)	4 (7.5)	**0.04**	0.06	**0.01**	0.5
β_2_GPI Phadia	3 (3.2)	8 (7.5)	7 (13.2)	0.07			

Occurrence of IgA, IgG and IgM anti-CL and anti-β_2_GPI were compared using three cut-offs: individual national cut-offs based on the 95th and 99th percentiles among national controls and cut-offs as recommended by the manufacturer. Statistical comparisons between each of the two groups were performed when the overall comparison showed significant difference. Significant *p*-values are depicted in bold.

β _2_GPI: β_2_ glycoprotein I; CL: cardiolipin; NP: non-pregnant; RD: recently delivered.

**Table 2:** Prevalence of anticardiolipin and anti–β_2_ glycoprotein I isotypes tested with particle-based multi-analyte technology among healthy NP, pregnant and RD Sudanese females.

	NP *n* = 92 *n* (%)	Pregnant *n* = 109 *n* (%)	RD *n* = 53 *n* (%)	*p*-value (overall)	*p*-value (NP-pregnant)	*p*-value (NP-RD)	*p*-value (pregnant-RD)
IgA							
CL 95th	4 (4.3)	55 (50.5)	26 (50)	**<0.0001**	**<0.0001**	**<0.0001**	0.9
CL 99th	0 (0)	37 (33.9)	15 (28.8)	**<0.0001**	**<0.0001**	**<0.0001**	0.5
CL Inova	3 (3.3)	47 (43.1)	23 (44.2)	**<0.0001**	**<0.0001**	**<0.0001**	0.9
β_2_GPI 95th	4 (4.3)	49 (44.9)	27 (51.9)	**<0.0001**	**<0.0001**	**<0.0001**	0.4
β_2_GPI 99th	0 (0)	37 (33.9)	16 (30.8)	**<0.0001**	**<0.0001**	**<0.0001**	0.7
β_2_GPI Inova	8 (8.7)	66 (60.5)	37 (71.1)	**<0.0001**	**<0.0001**	**<0.0001**	0.2
IgG							
CL 95th	4 (4.3)	5 (4.6)	2 (3.8)	0.9			
CL 99th	0 (0)	2 (1.8)	0 (0)	0.3			
CL Inova	1 (1.1)	3 (2.7)	1 (1.9)	0.7			
β_2_GPI 95th	4 (4.3)	2 (1.3)	1 (1.9)	0.5			
β_2_GPI 99th	0 (0)	1 (0.9)	0 (0)	0.5			
β_2_GPI Inova	2 (2.2)	1 (0.9)	1 (1.9)	0.7			
IgM							
CL 95th	4 (4.4)	5 (4.7)	4 (7.8)	0.6			
CL 99th	0 (0)	2 (1.9)	2 (3.9)	0.2			
CL Inova	0 (0)	1 (0.9)	1 (1.9)	0.4			
β_2_GPI 95th	4 (4.4)	9 (8.4)	6 (11.8)	0.3			
β_2_GPI 99th	0 (0)	1 (0.9)	2 (3.9)	0.1			
β_2_GPI Inova	0 (0)	1 (0.9)	2 (3.9)	0.1			

Occurrence of IgA, IgG and IgM anti-CL and anti-β_2_GPI were compared using three cut-offs: individual national cut-offs based on the 95th and 99th percentiles among national controls and cut-offs as recommended by the manufacturer. Statistical comparisons between each of the two groups were performed when the overall comparison showed significant difference. Significant *p*-values are depicted in bold.

β_2_GPI: β_2_ glycoprotein I; CL: cardiolipin; NP: non-pregnant; RD: recently delivered.

**Figure 3:**
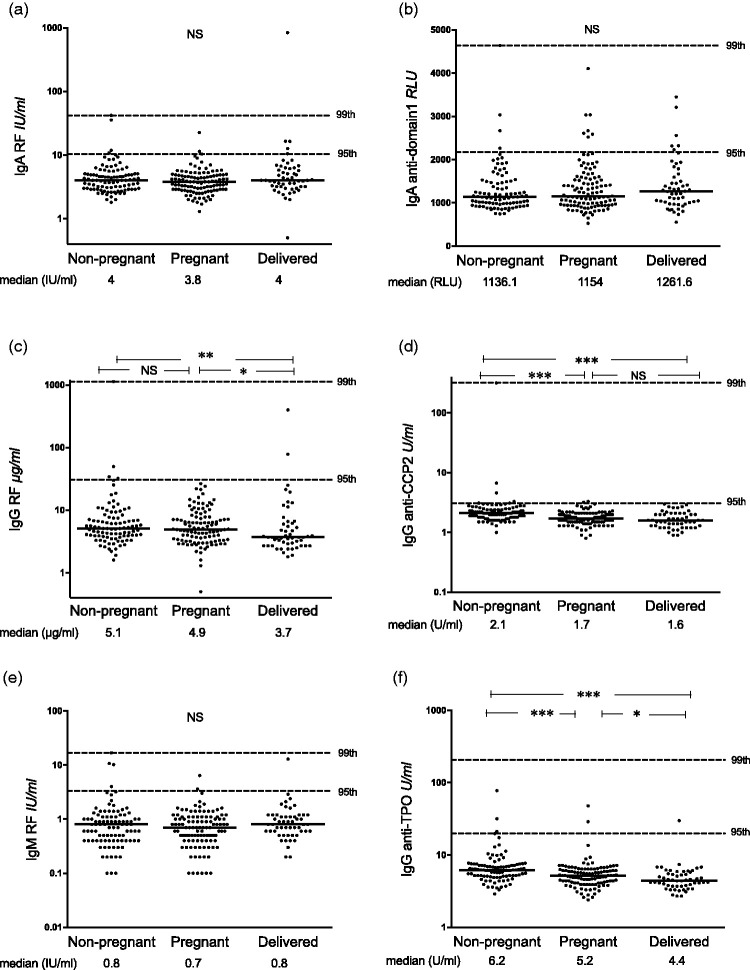
Levels of control antibodies and anti–β_2_ glycoprotein I domain 1 antibodies among Sudanese women. (a), (c) and (e) IgA/G/M rheumatoid factor (RF), (b) IgA anti–domain 1, (d) IgG anti–cyclic citrullinated peptide 2 (anti-CCP2) and (f) IgG anti–thyroid peroxidase (anti-TPO) among healthy non-pregnant, pregnant and recently delivered Sudanese females. Horizontal dotted lines represent the 95th and 99th percentiles among the healthy non-pregnant women. One healthy non-pregnant subject has anti-TPO outside the graph range, not shown in the figure but included in the statistics. Relative light unit (RLU) in panel (b) is a raw measurement unit and not a final calibrated unit. Comparisons between individual groups were made for those antibodies showing an overall significant difference between the three groups. *p < 0.05, **p < 0.01, ***p < 0.001, NS: not significant.

IgA and IgM RF did not differ between non-pregnant, pregnant and recently delivered women, whereas levels of IgG RF, IgG anti-CCP2 and IgG anti-TPO were lower in the pregnant and recently delivered groups (Figure 3(a), (c) to (f)). Levels of IgA antibodies against β_2_GPI-D1 did not differ between the groups (Figure 3(b)).

After stratifying the pregnant Sudanese women in relation to pregnancy duration at the time of blood sampling, IgM anti-β_2_GPI levels were higher during the second trimester compared with the first trimester (4.0 versus 2.3 ELIA U/ml, *p* = 0.007; FEIA); otherwise there were no differences for any aPL with both assays. Sudanese women’s parity did not show any associations to the levels of any aPL (data not shown).

**Figure 4:**
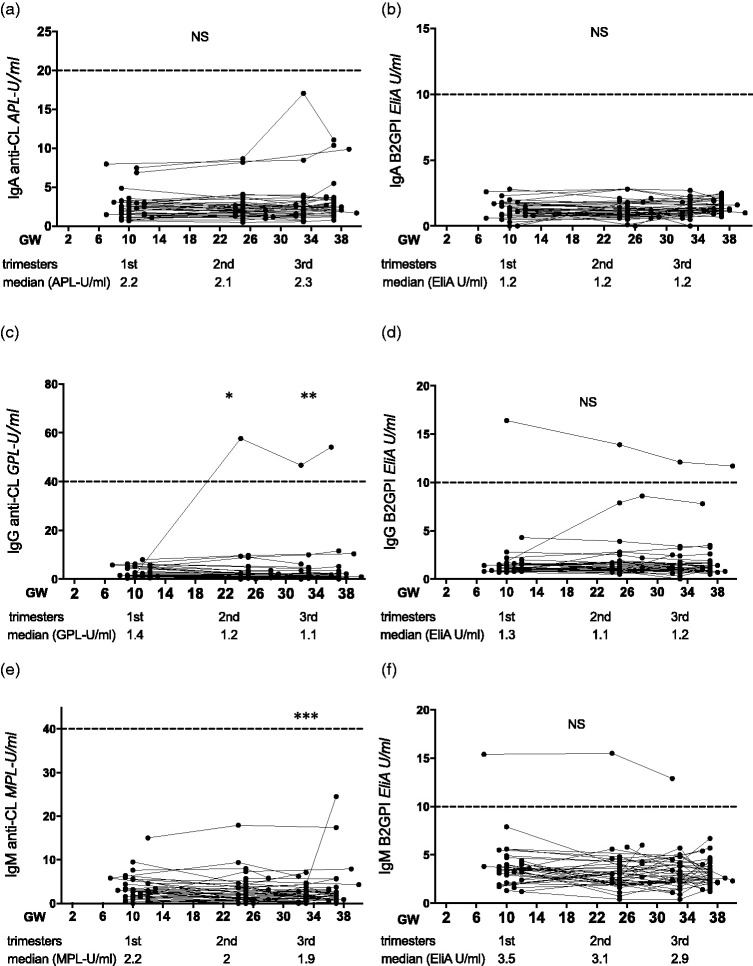
Serial measurements of IgA/G/M anticardiolipin (anti-CL) and anti–β_2_ glycoprotein I (anti-β_2_GPI) antibodies during normal pregnancies of Swedish women. (a), (c) and (e) IgA/G/M anti-CL and (b), (d) and (f) IgA/G/M anti-β_2_GPI levels among 42 healthy pregnant Swedish women investigated during the first, second, early third and late third trimesters that are represented in gestational weeks (GW). Below each panel, median antibody levels are shown for the first, second and early third trimester time points. Horizontal lines represent the cut-offs for clearly positive reactivity, as recommended by the manufacturer. Comparisons were made against first trimester. *p < 0.05, **p < 0.01, ***p < 0.001, NS: not significant.

Among the serially followed Swedish pregnant women, there were no fluctuations in IgA anti-CL or IgA, IgG and IgM anti-β_2_GPI during pregnancy; however, compared with the first trimester levels anti-CL dropped in the second and third trimesters for IgG and in the third trimester for IgM (*p* = 0.03 and *p* = 0.007 and *p* = 0.0006, respectively; Figure 4). Occurrence of aPL did not differ between Swedish pregnant women and Swedish non-pregnant controls; this concerned all isotypes and all trimesters investigated separately (data not shown).

## Discussion

In this study we detected elevated levels and increased occurrence of both IgA anti-CL and IgA anti-β_2_GPI among Sudanese pregnant women (15.9% and 32.7%, respectively). A similar trend was also seen for IgM anti-β_2_GPI using one assay. Such differences were not observed in Swedish pregnant women.

The number of non-pregnant Sudanese controls was limited (95 and 92 serum samples were available for FEIA and PMAT, respectively). Although these numbers are less than what is recommended by the International Society on Thrombosis and Haemostasis^35^ to calculate the 99th country-based cut-off for aPL, comparisons using the 99th cut-off among Sudanese women yielded similar findings as the 95th national cut-offs and company suggested cut-offs. It is noteworthy that previous studies have described low-titer aPL (>95th and <99th percentile) rather than high-titer (>99th) to be more clinically significant for obstetric APS.^36,37^

Prevalence of aPL in healthy asymptomatic individuals is 1–5% for anti-CL and 3% for anti-β_2_GPI.^38^ The predictive value for APS clinical events in asymptomatic carriers has been addressed by few studies. In an old study from 1996 on 1600 healthy pregnant women who were followed up during pregnancy and after delivery, the authors showed that women with IgG anti-CL had increased risk for pre-eclampsia, fetal growth retardation and fetal demise compared with sero-negative women.^39^ Lynch et al. also reported a similar conclusion, where high IgG anti-CL in early pregnancy could predict adverse pregnancy outcomes.^28^ Moreover, in agreement with these studies, Faden et al. demonstrated in a prospective cohort that IgG and/or IgM anti-β_2_GPI were associated with pre-eclampsia or eclampsia in healthy pregnant females even if other anti-CL isotypes were not present.^40^ Interestingly, Tortosa et al. recently reported that individuals with isolated IgA anti-β_2_GPI but without history of APS events had more APS-related events, mainly arterial thrombosis, during 5-year follow-up compared with individuals negative for aPL.^41^ However, it is noteworthy that risk stratification using the antiphospholipid score (aPL-S)^42^ and/or the global APS score (GAPSS)^43^ does not involve IgA aPL. These two scoring systems have been described as inconclusive for assessment of pregnancy morbidity risk.^44^ In the current study, for logistic reasons we were not able to obtain information regarding the outcome of pregnancies in Sudanese women. However, it was documented that all women had normal previous pregnancies that ended in delivering healthy live births.

IgA has been described to be the most prevalent aPL isotype in populations of African origin.^10,16–19^ In parallel, our group recently reported a predominance of IgA RF in Sudanese rheumatoid arthritis patients.^45^ In the current study, the high levels and prevalence of IgA anti-CL and anti-β_2_GPI in Sudanese pregnant females was not associated with increased IgA RF levels, arguing against a general IgA increase in these women but instead indicating an antigen-specific increase in IgA aPL. Interestingly, when comparing Swedish pregnant with non-pregnant women as well as serially investigating anti-CL and anti-β_2_GPI autoantibodies throughout Swedish normal pregnancies we could not observe increase in any aPL, including the IgA isotype. Thus, the increased IgA aPL levels seem to be characteristic for pregnancies in Sudan. We cannot from the current study disentangle if the origin is genetic or environmental. Genetic investigation of this Sudanese cohort is planned.

Our finding of lower levels of IgG autoantibodies in Sudanese pregnant compared with non-pregnant women as well as late drop of IgG aPL in serially followed pregnant Swedes agree with previous studies showing general decrease of total IgG during normal pregnancies,^22–24^ which can be explained by the concomitant physiological changes as plasma volume expansion and/or passage of IgG through the placenta to the fetus. Additionally, in the PROMISSE study on patients with SLE and/or APS, Yelnik et al. reported a decrease in IgG anti-CL and anti-β_2_GPI levels during the second and third trimester as compared with early pregnancy, although these changes in aPL levels did not predict pregnancy outcomes.^46^

To date, we do not know the clinical importance of this increase in IgA aPL during apparently healthy Sudanese pregnancies. In 2006, the worldwide stillbirth rate was calculated to be 23.9 stillbirths per 1000 deliveries, with the highest incidence in sub-Saharan Africa (32.2/1000) that is far higher than in the developed countries (5.3/1000).^47^ During 2010–2011, 2.16% (106/4895) of all deliveries at Soba University Hospital in Khartoum, Sudan resulted in stillbirths (Sahwa Elbagir, personal communication). It is important to find out if unrecognized increase in IgA aPL during pregnancy can explain part of these fatalities, in which case testing for IgA aPL during normal pregnancies would be of clinical importance. It is known from previous studies that persistently high aPL levels is associated with unfavorable pregnancy outcomes.^48^

The β_2_GPI molecule consists of five domains, where IgG and IgM antibodies against the outmost domain 1 are considered to be especially associated with pregnancy morbidity and fetal loss.^7,8,49,50^ After obtaining our results showing the striking IgA reactivity against CL and β_2_GPI in Sudanese pregnant women using one brand of reagents, the study was extended with repeated analyses of the conventional aPL including IgA antibodies specifically directed against β_2_GPI-D1, using reagents and technology provided by another company. The repeated analyses showed essentially identical results with both test systems, replicating the increase in IgA aPL and IgM anti-β_2_GPI in pregnant Sudanese women. However, we did not observe a corresponding increase in levels of IgA anti-β_2_GPI-D1 antibodies among the Sudanese pregnant women, and median levels were essentially similar among non-pregnant and pregnant women as well as those recently delivered. The literature on domain specificity for IgA anti-β_2_GPI autoantibodies is considerably scarce compared with conventional IgG and IgM autoantibodies. Despierres et al. reported that IgA antibodies against domain 4/5 associated with SLE without thrombosis whereas IgA anti-β_2_GPI-D1 was not associated with thrombosis or SLE.^51^ These data are corroborated by a recent report showing that IgA anti-β_2_GPI from patients with thrombotic APS do not mainly bind domain 1, but instead target three sites in domains 3, 4 and 5.^52^ Monoclonal antibodies against these epitopes have previously been shown to produce APS in animal models.^53^ Murthy et al. investigated three multi-ethnic cohorts and showed high correlation between IgA anti-β_2_GPI and IgA anti–domain 4/5 levels, as well as occurrence of thrombotic events and pregnancy losses in 77% of patients positive for anti-β_2_GPI domain 4/5 antibodies.^15^ Consequently, β_2_GPI-D1 might not be the major target for pathological IgA anti-β_2_GPI, and the non-reactivity against domain 1 of the aPL found in Sudanese pregnant women cannot be interpreted as a clear sign for non-pathogenicity of IgA aPL in this context. Further studies are warranted.

The current study is, to the best of our knowledge, the first report showing increased prevalence of IgA aPL in healthy pregnant African women, a finding that was not observed in the Swedish population. A limitation to this study is the cross-sectional design and the lack of follow-up of pregnancy outcomes and aPL levels which could have enabled us to stratify risk for maternal and fetal morbidity and mortality. Also, sampling was performed only once and not in agreement with the current Sapporo classification criteria for APS.^1^ As our current findings of increased IgA aPL during Sudanese normal pregnancies were unexpected, we regard this study as hypothesis generating. To understand if and when during pregnancy IgA aPL should be investigated, and whether single or repeated testing may be needed, asymptomatic pregnancies in Sudanese women should be followed serially both concerning aPL levels and clinical outcome.
